# Malaria predictions based on seasonal climate forecasts in South Africa: A time series distributed lag nonlinear model

**DOI:** 10.1038/s41598-019-53838-3

**Published:** 2019-11-29

**Authors:** Yoonhee Kim, J. V. Ratnam, Takeshi Doi, Yushi Morioka, Swadhin Behera, Ataru Tsuzuki, Noboru Minakawa, Neville Sweijd, Philip Kruger, Rajendra Maharaj, Chisato Chrissy Imai, Chris Fook Sheng Ng, Yeonseung Chung, Masahiro Hashizume

**Affiliations:** 1 Department of Global Environmental Health, Graduate School of Medicine, The University of Tokyo, Tokyo, Japan; 2 Application Laboratory, Japan Agency for Marine-Earth Science and Technology, Yokohama, Japan; 3 Institute of Tropical Medicine, Nagasaki University, Nagasaki, Japan; 4 Alliance for Collaboration on Climate and Earth Systems Science, Cape Town, South Africa; 5 Department of Health, Limpopo, South Africa; 6 Office of Malaria Research, Medical Research Council, Durban, South Africa; 7 Australian Institute of Health Innovation, Macquarie University, Sydney, Australia; 8 School of Tropical Medicine and Global Health, Nagasaki University, Nagasaki, Japan; 9 Department of Mathematical Sciences, Korea Advanced Institute of Science and Technology, Daejeon, Republic of Korea

**Keywords:** Projection and prediction, Epidemiology, Epidemiology

## Abstract

Although there have been enormous demands and efforts to develop an early warning system for malaria, no sustainable system has remained. Well-organized malaria surveillance and high-quality climate forecasts are required to sustain a malaria early warning system in conjunction with an effective malaria prediction model. We aimed to develop a weather-based malaria prediction model using a weekly time-series data including temperature, precipitation, and malaria cases from 1998 to 2015 in Vhembe, Limpopo, South Africa and apply it to seasonal climate forecasts. The malaria prediction model performed well for short-term predictions (correlation coefficient, r > 0.8 for 1- and 2-week ahead forecasts). The prediction accuracy decreased as the lead time increased but retained fairly good performance (r > 0.7) up to the 16-week ahead prediction. The demonstration of the malaria prediction process based on the seasonal climate forecasts showed the short-term predictions coincided closely with the observed malaria cases. The weather-based malaria prediction model we developed could be applicable in practice together with skillful seasonal climate forecasts and existing malaria surveillance data. Establishing an automated operating system based on real-time data inputs will be beneficial for the malaria early warning system, and can be an instructive example for other malaria-endemic areas.

## Introduction

Malaria is a life-threatening infectious disease caused by parasites transmitted to human through a mosquito vector. Effective malaria control strategies have decreased the burden of malaria in recent decades. Globally, an 18% reduction in malaria incidence was estimated from 2010 to 2017^1^. However, there is still much uncertainty in the decline of the malaria burden because of spatial heterogeneity and temporal change^2^, and a high burden is still being reported. In 2017, global estimates of 219 million new malaria cases and 451,000 deaths by malaria were reported^1^.

The World Health Organization (WHO) African Region was estimated to have the most substantial burden of malaria, accounting for 92% of malaria cases in 2017^1^. In South Africa, malaria cases have occurred predominantly in three provinces in the north and northeast (KwaZulu-Natal, Limpopo, and Mpumalanga) near the borders with Mozambique, Zimbabwe, and Botswana. The South African government has the aim of eliminating malaria by 2020, which is a common goal among the Elimination 8 Regional Initiative countries^3^. The number of malaria cases in South Africa decreased gradually from 8,060 in 2010 to 1,157 in 2015, but resurged in 2016 and reached 22,517 cases in 2017^1^.

Malaria is sensitive to climate. Rainfall and temperature are considered the main weather factors that highly determine malaria epidemics^4^. High rainfall increases the number of breeding sites for mosquitoes and leads to increases in malaria transmission. Some studies, however, have reported that intense rainfall could flush early-stage larvae and shrink mosquito populations in the short term^5^. High temperatures increase the chance of transmission by shortening the duration of parasite growth in mosquitoes^6^. Temperature changes also influence the development, reproduction, survival, and biting rate of mosquitoes^7–9^.

A malaria early warning system (MEWS) has been considered as a promising tool to prevent/reduce the burden of malaria by accounting for the complexities in the malaria–climate dynamics often changing unexpectedly. Basic elements for a MEWS should include case surveillance (early detection of epidemics), an early warning based on meteorological conditions, and seasonal climate forecasts^10^. A robust MEWS not only requires linking the basic elements such as the monitoring/predicting information described above, but should also be based on the high quality of elements that are expected to be persistent and well-organized. In addition, a malaria prediction model that performs accurately together with a high quality malaria surveillance system and high quality seasonal climate forecasts is required to establish a successful MEWS.

Although several MEWSs have been developed^11,12^, no sustainable system has remained because of several operational difficulties. Previous studies have proposed malaria forecasting methods based on different modeling approaches such as statistical modeling (e.g., generalized linear model (GLM) and autoregressive integrated moving average (ARIMA) time series model), mathematical modeling (e.g., susceptible-exposed-infected-recovered (SEIR) model), and machine learning methods (e.g., neural network)^13^. However, no one method has been a gold standard because each method has different modeling assumptions and the optimal choice of the method depends upon the characteristics of a study population. Another challenge to implement a MEWS in practice may be the great computational burden for obtaining seasonal climate forecasts periodically. Only few studies, for example, in Botswana, West Africa, and India have attempted to demonstrate malaria forecasting by integrating their malaria prediction models with seasonal hindcasts of meteorological variables^14–16^.

In South Africa, the malaria surveillance system has been operating effectively over the past few decades^17^, as malaria is one of notifiable diseases. Although South Africa is regarded as a hypo-endemic malaria area, the relatively well-established malaria surveillance system could be an instructive example to develop a malaria prediction model for use in a MEWS. In this study, we accessed the malaria surveillance database in Vhembe, Limpopo Province to develop a weather-based malaria prediction model for the area, which accounts for more than 60% of the malaria burden in South Africa^18^. We applied a flexible statistical modeling approach, a GLM with a distributed lag nonlinear structure, to understand the complexity of nonlinear and delayed malaria-weather associations and develop a weather-based malaria prediction model accordingly. We also demonstrated the malaria prediction model performance based on seasonal climate forecasts to determine if the prediction model could feasibly be used in practice.

## Methods

### Malaria surveillance data

We obtained malaria data of patients in Vhembe, Limpopo, South Africa from 1 January 1998 to 31 May 2015 that were collected by the surveillance system of the Limpopo Department of Health Malaria Program. All malaria cases recorded in the surveillance system were based on positive blood test results, either through microscopy or a malaria rapid diagnostic test. Clinically diagnosed cases without a positive blood test were excluded from the surveillance system. The surveillance data also included demographic characteristics (e.g., sex and age), date of each case reported, type of malaria parasite, and survey type (passive notification or active survey). The active survey was implemented for the targeted villages only if a malaria case was reported, so makes up only 5% of the surveillance data.

### Meteorological observed reanalysis data

We considered two meteorological variables for use in the malaria prediction model: (1) precipitation and (2) ambient temperature. The daily time series of the meteorological variables for the same period as the malaria data were obtained from sources that could provide the data almost in real-time. The observed or estimated daily precipitation was derived from the African Rainfall Climatology, version 2 (ARC2) dataset that combines data from the National Oceanic and Atmospheric Administration (NOAA)/National Center for Environmental Prediction (NCEP)/Climate Prediction Center (CPC) Famine Early Warning System Network (FEWS-NET)^19^. The data can be downloaded from https://iridl.ldeo.columbia.edu/SOURCES/.NOAA/.NCEP/.CPC/.FEWS/.Africa/.DAILY/.ARC2/.daily/index.html. The horizontal resolution of the precipitation was 0.1° × 0.1° (about 10 km × 10 km). We also obtained the 2-m ambient temperature observed/estimates from the NCEP-National Center for Atmospheric Research (NCAR) reanalysis^20^, which is available at https://www.esrl.noaa.gov/psd/data/gridded/data.ncep.reanalysis.html. The horizontal resolution of the temperature was 2.5° × 2.5° (about 250 km × 250 km). We averaged the precipitation and the 2-m air temperature over the regions covered in our study area (22.3°S to 23.0°S and 29.2°E to 30.6°E).

### Retrospective seasonal climate forecasts

The retrospective seasonal climate forecasts for daily precipitation and 2-m ambient temperature across the regions described above were derived from the dynamically downscaled forecasts of the Scale Interaction Experiment-Frontier Research Center for Global Change, version 2 (SINTEX-F2) Coupled ocean–atmosphere General Circulation Model (CGCM). The data were obtained for the sub-annual period of July–April over the whole study period (1998–2015), which covered endemic malaria seasons in the study area. We used the Weather Research and Forecasting (WRF) model for the dynamical downscaling^21^ so that the horizontal resolution of the SINTEX-F2 CGCM forecasts at 1.1° × 1.1° (about 120 km × 120 km) was downscaled at 9 km x 9 km. We also corrected the systematic bias in simulating atmospheric circulation before implementing the dynamical downscaling^22^ according to previous studies that demonstrated improved SINTEX-F2 forecasts in the southern African region^23,24^. The SINTEX-F2 system can predict climate variability not only in the tropical Pacific but also in tropical and subtropical Indian Ocean, which can work as potential sources of seasonal predictability over southern Africa. Details of the SINTEX-F2 CGCM forecasts (e.g., model set-up, forecasting experiments, and prediction skills) have been described elsewhere^25,26^.

### Statistical analysis

We aggregated the datasets described above (i.e., individual malaria surveillance data, the daily time series of the weather observations, and the seasonal climate forecasts) to weekly time series for statistical analysis. To obtain the same number of weeks every year (1–52 weeks), we constructed the weekly data starting on 1 January for each year, so the last 1–2 days corresponding to the 53^rd^ week were excluded from our analyses.

The statistical analysis had three main parts: (1) model building to understand the nonlinear and delayed malaria-weather associations and develop a weather-based malaria prediction model accordingly, (2) testing the prediction model based on the reanalysis weather observations assuming perfect weather forecasts, and (3) demonstrating the prediction process based on the bias-corrected and downscaled SINTEX-F2 seasonal climate forecasts appended to the most recent available weather observations.

### Model building

We fitted a GLM with a Poisson family taking into account over-dispersion. We considered a distributed lag nonlinear structure to investigate the nonlinear and nonlinearly-delayed associations between malaria incidence and weather factors^27^. The distributed lag nonlinear model (DLNM) has been used widely in studies that examined the association between environmental exposure and health outcome, for example, the temperature-mortality association^28^ and the climate-dengue association^29^. Specifically, we incorporated a cross-basis function for the temperature-malaria association in the model, characterized by a quadratic B-spline with an internal knot at the 50^th^ percentile of temperature and a natural cubic spline for a lag of 0–12 weeks with two internal knots equally-spaced on a log scale. We chose the maximum lag of 12 weeks using the lag-response curve for the temperature–malaria association in which the period of 0–12 weeks was long enough to capture the increased risk of malaria for temperature as well as the decreased displacement pattern of the risks.

To examine the association between precipitation and malaria incidence, we used a simpler approach than the distributed lag nonlinear structure to minimize the correlation between temperature and precipitation terms that tended to result in high uncertainty in malaria predictions. We also considered different lag periods of precipitation under the hypothesis that short and long delayed effects of precipitation may have independent roles. We applied two nonlinear functions of the short- and long-term averages of precipitation (from the current week to the preceding 15 weeks and 51 weeks, respectively) using a natural cubic spline with 5 and 3 degrees of freedom, respectively, after a natural log transformation due to the skewness of the distribution.

We also added a linear term of the previous malaria cases at the preceding week after transforming it by a natural logarithm. This additional term is regarded as a risk factor based on the general feature of infectious disease (i.e., the vector–host–vector transmissions in our study), and incorporating the term could reduce the serial auto-correlation in the residuals of the model^30^. Another linear term of the year was also included in the model to adjust for the long-term declining trend of malaria incidence. The model framework and equations are provided in the Supplementary Information. The modeling choices were tested in sensitivity analysis, described in the separate section below.

From the model fit, we estimated the associations between malaria incidence and weather factors as a relative risk (RR) indicating a risk for the exposure levels compared with that at a reference value. In our study, we set the reference point as the values representing the minimum estimated risk of malaria incidence for temperature and 1-year average of precipitation (i.e., 12.1 °C and 2.0 on the log scale, respectively). We also used zero (log scale) as a reference for the 4-month average precipitation.

We compared four models including a different combination of the covariates as follows: Model 1, temperature only; Model 2, two covariates representing short- and long-term precipitations; Model 3, temperature and the two precipitation terms; and Model 4, addition of the previous malaria cases to Model 3.

### Testing the prediction model based on the weather observations

Using the DLNM, we predicted the (retrospective) future number of malaria cases for 1–16 weeks to evaluate the model’s prediction performance. We used the observed temperature and precipitation data as the forecast information (perfect forecast) to examine the performance to avoid involving the uncertainty of the climate prediction. We employed a self-updating process to generate values for the auto-correlation terms in the model. Specifically, we predicted the number of malaria cases one week ahead using the observed weather data. This first 1-week-ahead prediction of malaria cases was then regarded as the auto-correlation term in the subsequent model to obtain the second 1-week-ahead prediction of malaria cases. Next, the first and second 1-week-ahead predictions were used to compute the third 1-week-ahead prediction, and so on until the sixteenth 1-week-ahead prediction was obtained.

To demonstrate a real-time weekly-updated prediction process, we used subsets of data (9 years; approximately 50% of the data) to build the DLNM and obtain the (retrospective) future malaria prediction for the forecasting periods (the following 1–16 week). We moved the 9-year data window forward week by week, which generated a total of 416 iterations (Supplementary Fig. S1).

### Accuracy of the malaria prediction models

We used several measures to assess the accuracy of the malaria prediction model. First, we calculated the Spearman correlation coefficient between the observed and predicted malaria cases. Second, we calculated the root mean square error (RMSE); the equation of the RMSE is described in the Supplemental Information. Third, we calculated the area under the curve (AUC) of the receiver operating characteristic (ROC). To do this, we defined an outbreak of malaria using a level of the threshold at the 40^th^ percentile of the past moving 5-years malaria cases during the endemic season (September–May). We chose the threshold level of the 40^th^ percentile by taking into account the AUC values by different levels of the threshold from the 40^th^ to the 80^th^ percentiles (Supplementary Fig. S2). Forth, we calculated a concordance rate (%), defined by the ratio of the sum of true positive and true negative values (weeks) to the total number of weeks. Lastly, we calculated sensitivity and specificity. The sensitivity was defined as the ratio of the number of predicted outbreaks to the number of true observed outbreaks. The specificity was defined as the ratio of the number of predicted non-outbreaks to the number of true observed non-outbreaks. All the accuracy measures were calculated for each lead time of 1–16 week ahead over the iterations described in the previous section above.

### Demonstrating the prediction model based on seasonal climate forecasts

To demonstrate the implementation of the malaria prediction model in a real setting, we modified the weekly-updated prediction process described above by replacing the observed weather data with the seasonal climate forecasts produced by the dynamical downscaled SINTEX-F2. Because we considered the delayed effects of temperature and precipitation in the DLNM, we used a hybrid of weather data consisting of observed values and climate forecasts at a certain time point for the malaria predictions. Also, although the observed weather data can be provided in almost real time, we supposed that the seasonal climate forecasts could be updated only once a month because of the heavy computational cost. Thus, the same climate forecast data were used for the weekly-updated malaria predictions (retaining the same lead time of 1–16 weeks) until the following month when the next updates of climate forecasts were provided. The time periods of this test were shorter from July to April than for the time periods that used the observed weather data because of the availability of the seasonal climate forecasts.

### Sensitivity analysis

We altered the DLNM specification and chose the best models with higher levels of prediction performance using the accuracy measures described above: correlation coefficients, RMSE, concordance rate, and the AUC (Supplementary Fig. S3). First, we changed the degree of freedom (df) for the associations between malaria incidence and the short- and long-term averages of precipitation from 2 to 6 and from 1 to 5, respectively. Second, we changed the number of internal knots for the temperature–malaria association from 1 to 3. Third, we changed the functional form of the auto-correlation terms: (1) a single lag of preceding week as a linear term, (2) a single lag of preceding week as a natural cubic spline with 3 df, (3) a moving average of preceding 1–4 weeks as a natural cubic spline with 3 df, and (4) a cross-basis function including a quadratic B-spline with a knot at the 50^th^ percentile of the preceding 1–4 weeks and a natural cubic spline for the nonlinear distributed lags with a knot at equally-spaced log value. Lastly, we changed the lag periods of the short-term average of precipitation from 0–4 weeks to 0–24 weeks.

All analyses were performed using R version 3.5.1 with the packages “dlnm” for the distributed lag nonlinear models and “pROC” for the AUC calculations.

## Results

A total of 53,689 patients who were diagnosed with malaria were included in the present study. Mean age of the patients was 25.2 years (standard deviation (SD) = 17.7); 55.2% were male and 44.8% were female. The *Plasmodium falciparum* was predominant among the infected patients (99.6%). It was identified by a definitive diagnosis using either a malaria rapid diagnostic test or a microscope blood smear. The fatality rate was 1.0% (534 patients).

**Figure 1:**
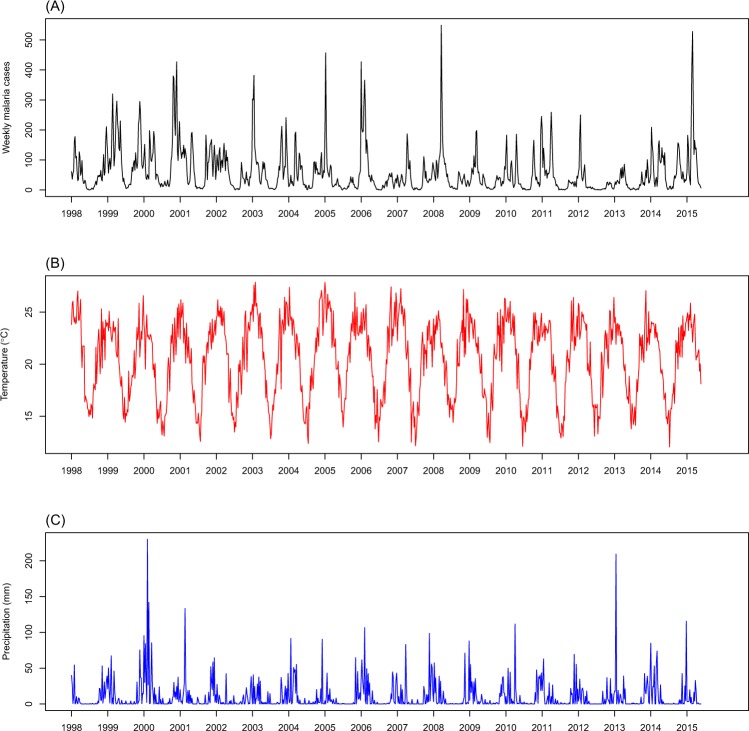
Weekly time series of (**A**) malaria cases, (**B**) mean 2-m air temperature (°C), and (**C**) total precipitation (mm) in Vhembe district, Limpopo Province, South Africa.

Figure 1 shows the weekly time series of malaria patients, mean 2-m air temperature, and total precipitation in the study area. The average weekly number of patients with malaria was 59 (SD = 72), and, in general, the numbers decreased over time. The malaria incidence also showed clear seasonal fluctuations with higher levels in warm and rainy seasons (average = 76 and SD = 75 during September–May) and lower levels in cool and dry seasons (average = 7 and SD = 8 during June–August). The average weekly temperature was 20.6 °C (SD = 3.7 °C) over the years, and it ranged from 12.1 °C to 27.9 °C. The average weekly total precipitation was 9.7 mm (SD = 20.5 mm); however, the distribution of the precipitation was skewed because of heavy rainfalls, particularly in 1999–2000 and 2012–2013.

### Associations of malaria incidence with weather factors

**Figure 2:**
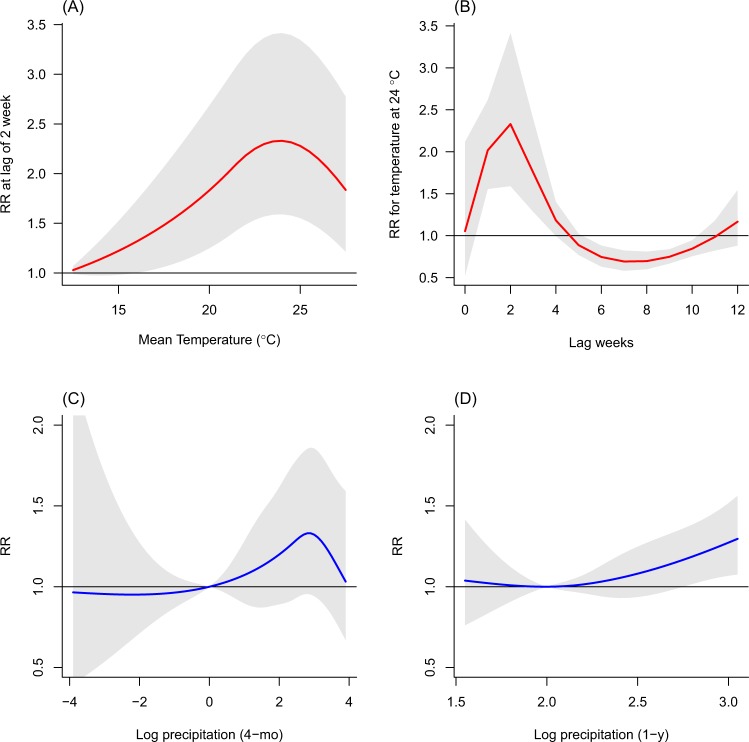
Associations between malaria incidence and weather factors. The relative risks (RR) for temperature (**A**) at lag of 2 weeks and (**B**) the lag-response curve for 24 °C, and the RR for the log precipitation of (**C**) 4-month (4-mo) and (**D**) 1-year (1-y) averages.

Figure 2 shows the associations between weather factors and malaria incidence. For the association with temperature, nonlinear and delayed patterns were obvious over the 0–12 lag weeks adjusting for precipitation (see also the 3D plot in Supplementary Fig. S4). Specifically, we found that the relative risk (RR) for temperature at lag of 2 weeks increased with increasing temperature, but reached the highest level of RR at moderate temperatures (approximately 23–24 °C) and then decreased at higher temperatures (Fig. 2A). The RR for 24 °C was highest at lag of 2 weeks (Fig. 2B).

The shape of curves for precipitation varied between the 4-month and 1-year averages (Fig. 2C,D). The RR for the 4-month average of precipitation increased as the weekly total precipitation increased up to approximately 20 mm (equal to 3 on the log scale) (Fig. 2C), then decreased at higher precipitation, which may be explained by the washing out the breeding sites of mosquitoes by heavy rainfalls. The washing-out effect may be temporary during a couple of months because, for the higher levels of the long term 1-year average of precipitation, the malaria incidence increased (Fig. 2D).

### Accuracy of the prediction models

**Figure 3:**
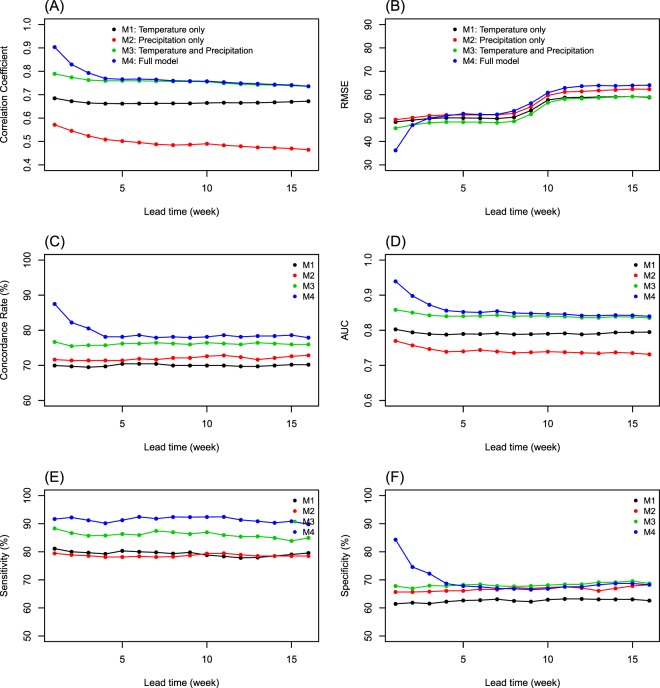
Prediction accuracy over lead time (week) using four different models. Model 1 (M1), temperature only; Model 2 (M2), short- and long-term precipitations only; Model 3 (M3), temperature and two precipitations; and Model 4 (M4), all variables with addition of the previous malaria cases from the weekly-updated malaria prediction process based on the observed weather data (assuming perfect weather forecasts). The accuracy measures were (**A**) Spearman correlation coefficient, (**B**) root mean square error (RMSE), (**C**) concordance rate (%), (**D**) area under the curve (AUC) of the receiver operating characteristic (ROC), (**E**) sensitivity (%), and (**F**) specificity (%).

Figure 3 shows the malaria prediction accuracy over the lead time among Models 1–4 from the weekly-updated prediction process using the observed weather data (assuming perfect weather forecast) for each of the accuracy measures (Spearman correlation coefficient, RMSE, concordance rate, AUC, sensitivity, and specificity). We found that Model 1 with the cross-basis function of temperature over the lag of 0–12 weeks captured the seasonal fluctuations of malaria transmission fairly well. However, the prediction accuracy was much improved after adding the 4-month and 1-year averages of precipitation to the model (Model 3). In Model 4, adding the auto-correlation term generally improved the short-term predictions for the 1–3 week lead time, particularly in the results of correlation coefficient, concordance rate, and AUC, although it showed slightly higher RMSE and lower specificity for longer-term predictions. We chose Model 4 as the best model to demonstrate the weekly-updated prediction process based on seasonal climate forecasts.

### Malaria predictions based on the seasonal climate forecasts

**Figure 4:**
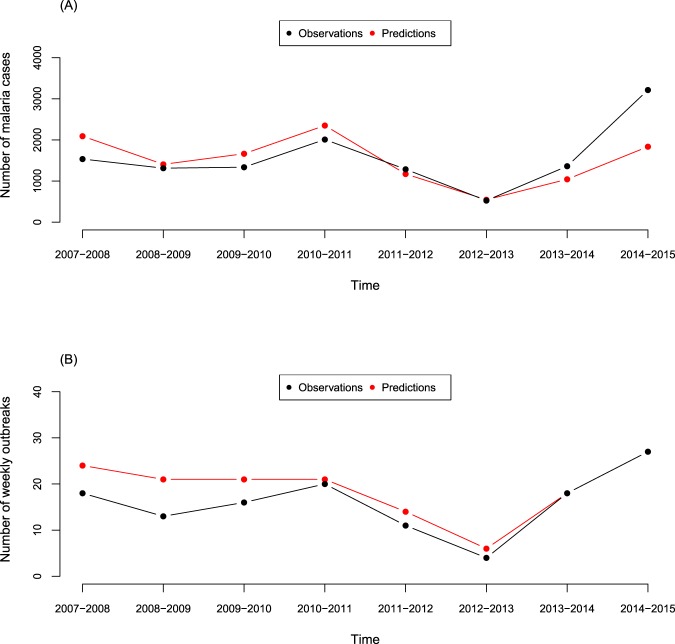
Cumulative annual number of malaria incidence and malaria outbreaks, defined by the 40^th^ percentile of the past moving 5-years malaria cases during the endemic season (September–May), for the 2-weeks-ahead lead time (red, predictions; black, observations) in the demonstration of the weekly-updated malaria prediction process based on seasonal climate forecasts.

Figure 4 shows the cumulative annual number of malaria incidences and malaria outbreaks for the 2-weeks-ahead lead time, selected from among the 1- to 16-weeks-ahead predictions. Overall, the 2-weeks-ahead prediction values coincided closely with the observed values, although they tended to be higher at the first four years out of eight years (Fig. 4A). The biggest gap between prediction and observation was during the 2014–2015 season, probably because the prediction model did not detect the huge outbreak in early 2015 (Supplementary Fig. S5-A). The trend of the number of outbreaks was consistent with the number of malaria incidences in general (Fig. 4B). However, these results somewhat varied depending on how to define the outbreak (Supplementary Fig. S6). We also provided the additional results for the 4-weeks-ahead predictions and observations in Supplementary Figs. S5-B, S7.

## Discussion

In this study, we developed a weather-based malaria prediction model using a flexible statistical approach that takes into account the nonlinear and delayed associations between malaria transmission and climate factors. The malaria prediction model showed good performance particularly for short-term predictions for 1- and 2-weeks ahead. The accuracy of the prediction decreased as the lead time increased, but retained fairly good performance up to 16-weeks ahead.

In the demonstration of the weekly-updated malaria prediction process using the dynamically downscaled seasonal climate forecasts, we also found that the short-term prediction values coincided closely with the observed values. To our best knowledge, a limited number of previous malaria prediction models used the seasonal climate forecasts that are required to implement such models in a real setting together with the malaria surveillance data^14–16^. The weekly-updated malaria prediction process demonstrated in our study would be merited if it is implemented in the local communities in Limpopo province, South Africa, which could provide better opportunities for public health practice such as improving decision making for the optimal deployment of resources (e.g., timely distribution of drugs and/or higher impact of malaria control interventions by more efficient scheduling of indoor residual spraying)^1^.

Our results for the association with temperature showed that the malaria risk was highest at moderate temperatures (approximately 23–24 °C as a weekly average) at lag of 2 weeks and decreased at lower or higher temperatures, although the risk was higher at extremely high temperatures (27.9 °C) than at extremely low temperatures (12.0 °C). Previous studies suggested there may be a critical temperature range for mosquito survival, and the longevity of mosquito vectors could be shortened at extremely high temperatures^7–9,31^. In our study, we observed a clear lag pattern for the malaria–temperature association. Significant higher risk persisted during lag of 1–4 weeks with the peak at lag of 2 for the moderate temperature (24.0 °C), followed by lower risk at lag of 6–10 weeks. The higher risk at the shorter lag periods seem reasonable when taking into account the overall lengths of the lifecycle of the malaria parasite (particularly for the incubation period in a human host) and gonotrophic cycle for vector mosquitoes. It is known that the incubation period from malaria infection by mosquito bite (sporozoites injected to human host) to when symptoms appear is an average of 12–14 days^32^. The gonotrophic cycle takes a few days but is highly dependent on the temperature; high temperatures can shorten the cycle and lead to an increase in the mosquito population^33^. The lower risk at longer lag periods seem to reflect the ‘harvesting’ phenomenon, an epidemiological term that describes the displacement of mortality after episodes of high temperature that temporarily reduce susceptible populations^34^. This could be explained by changes in the human host population attributable to the large risk increases in the early period of our study. However, the mechanism for the longer lag temperature effects on malaria based on epidemiological evidence is unclear. Different mechanisms besides displacement may exist to explain the harvesting-like lag pattern.

The nonlinear associations for precipitation were investigated using smooth functions of the average of preceding 4-month and 1-year weekly totals (reflecting sub-seasonal and longer-term precipitations, respectively). These smooth functions were simpler than the cross-basis function applied to temperature in terms of the number of parameters accounting for the exposure-response association and corresponding lag structure simultaneously. In general, evaluating the influence of precipitation on vector lifecycle is more variable and more uncertain than evaluating it with the temperature effects^33^. Also, the uncertainty of the seasonal climate forecasts for precipitation is generally larger than for temperatures^25,35^. We believe that our simpler approach could be beneficial to reduce the uncertainties potentially added up by different sources (i.e., the association for precipitation and the predicted precipitation described above) in the process of predicting malaria incidence. Nevertheless, we considered two different time scales of the precipitation to take into account the complex dynamic of mosquito populations associated with precipitation and thus their breeding sites as much as possible. The shapes of the curves for the sub-seasonal and longer-term precipitation varied considerably (Fig. 2C,D). The results suggest that persistence of breeding sites is less likely with high amount of precipitation caused by frequent heavy rainfalls within a season^5,36^, whereas higher annual precipitation in a preceding year may influence the hydrological environment overall in terms of the number of breeding sites, possibly related to soil moisture and vegetation in a longer-term perspective^37^. A previous modeling study reported that annual precipitation accounted for only 60% of the variance in adult mosquito populations in a Niger Sahel village in Africa, and the mosquito abundance was better explained by incorporating intra-seasonal rainfall variability and the simulated surface area of standing water than by annual precipitation alone^36^. Also, a 21-years cohort study using the Health and Demographic Surveillance System (HDSS) data in Agincourt in South Africa, which is located in the neighboring province of our study location (approximately 350 km away), reported that the increased risk of malaria mortality was significantly associated with both higher yearly rainfall and the joint effects of monthly rainfall–temperature, which adjusted each other in the model^38^.

Although our prediction model showed good performance for short-term malaria predictions in general, we recognized that the predictions incorporating the seasonal climate forecasts were largely underestimated for the 2014–2015 season, even the 2-weeks-ahead prediction results (Fig. 4A). The low accuracy for this season was due mainly to the unexpected huge outbreak that occurred during the 8^th^–10^th^ weeks in early 2015 (418, 528, and 291 cases, respectively). The precipitations did not seem to greatly contribute to this huge outbreak because the cumulative precipitation totals before the outbreak were lower (245.4 mm from the 35^th^ week of 2014 to the 7^th^ week of 2015) than the average in previous years (335.4 mm for the same week periods since the 2000–2001 season). Some unmeasured/unknown factors other than weather conditions might have played a role to facilitate the malaria transmission in early 2015. In Vhembe, the local cases have been predominant for the malaria burden^18^, which was observed consistently in our study in general (Supplementary Fig. S8). However, there was a sudden increase in the proportion of imported malaria cases during the two preceding seasons, 2012–2013 and 2013–2014. We speculate that an increase in the movement of people from areas with higher malaria prevalence outside of the country into Vhembe may have increased the risk of malaria transmission in the 2014–2015 season. Future studies, in collaboration with neighboring countries, to investigate how the interplay between the cross-border transmission and climate factors works are warranted to ultimately achieve the goal of the Elimination 8 Regional Initiative countries^3^.

We acknowledge some limitations in this study. First, we considered two weather factors (temperature and precipitation) and malaria transmission at a preceding week as only factors taken into account in our prediction model because of data availability of the seasonal climate forecasts, although other climatic factors such as relative humidity and normalized difference vegetation index (NDVI) have been used in the previous studies aiming to develop a malaria prediction model^13^. Second, we did not incorporate the potential role of temporal changes in the immunity levels to malaria when developing our prediction model, which may help to improve the malaria predictions in future studies. Third, our seasonal climate forecasts by the climate prediction models (SINTEX-F2) included some uncertainties that may have produced misclassification of the weather exposure to malaria cases. Further improvement of the seasonal forecast system (e.g., initialization, prediction modeling, and ensemble techniques) may generate better inputs for our malaria prediction model, and consequently, the malaria predictions could be more accurate in the future.

Some previous studies have proposed different modeling approaches^13^ for weather-based malaria forecasting such as statistical modeling (e.g., GLM, generalized additive model (GAM)^39^, and ARIMA model^40^), mathematical modeling (e.g., SEIR model), and machine learning methods (e.g., neural network^41^). Each model includes different assumptions and has its advantages and disadvantages. Statistical models such as GLM or GAM have greater interpretability regarding the relationship between weather factors and malaria but may not fully capture the residual serial correlations. The ARIMA model can represent the features of temporal patterns (i.e., seasonality and autocorrelation) more sophisticatedly but lacks interpretability of weather covariates. Mathematical models are based on a different assumption that individuals are transferred over time among separate compartments of susceptible, exposed, infected, and recovered groups. Although they represent the dynamics of malaria transmission more explicitly, their disadvantages include the difficulty in specifying key parameters, given the nature of deterministic (not stochastic) models and computational complexity. Lastly, the machine learning approach has been useful as it can handle the highly complex relationship with predictors but has suffered from overfitting and poor interpretability^13^. When performing malaria forecasting, the modeling choice should be guided by the assumptions of different methods and the characteristics of a study population. In this research, we adopted a GLM with a distributed lag nonlinear structure to ensure both interpretability and flexibility to describe the nonlinear and nonlinearly-delayed association between weather factors and malaria. To handle the residual correlation issue, we incorporated the auto-correlation term into the prediction model^30^. In future studies, a comparison of our forecasting method with other methods is warranted, and an ensemble of multiple predictions may achieve the best prediction performance.

In summary, we developed a malaria prediction model taking into account nonlinear and delayed complexities of the malaria–climate dynamics. The prediction model showed good performance for the short-term lead time, and the prediction accuracy decreased as the lead time increased but retained fairly good performance. We also demonstrated the weekly-updated malaria prediction process based on seasonal climate forecasts and found that the malaria predictions for short-term lead time coincided closely with the observed malaria cases. The malaria prediction model we developed is promising because it is feasibly applicable in practice together with the skillful seasonal climate forecasts and existing malaria surveillance system in South Africa. Establishing an automated operating system based on real-time data inputs could potentially be beneficial for the malaria early warning system in Limpopo, South Africa, and can be an instructive example for other malaria-endemic areas.

## Supplementary information

Supplementary information

Supplementary Fig. S1
