# Publisher Correction: Malaria predictions based on seasonal climate forecasts in South Africa: A time series distributed lag nonlinear model

**DOI:** 10.1038/s41598-020-58890-y

**Published:** 2020-02-05

**Authors:** Yoonhee Kim, J. V. Ratnam, Takeshi Doi, Yushi Morioka, Swadhin Behera, Ataru Tsuzuki, Noboru Minakawa, Neville Sweijd, Philip Kruger, Rajendra Maharaj, Chisato Chrissy Imai, Chris Fook Sheng Ng, Yeonseung Chung, Masahiro Hashizume

**Affiliations:** 1 Department of Global Environmental Health, Graduate School of Medicine, The University of Tokyo, Tokyo, Japan; 2 Application Laboratory, Japan Agency for Marine-Earth Science and Technology, Yokohama, Japan; 3 Institute of Tropical Medicine, Nagasaki University, Nagasaki, Japan; 4 Alliance for Collaboration on Climate and Earth Systems Science, Cape Town, South Africa; 5 Department of Health, Limpopo, South Africa; 6 Office of Malaria Research, Medical Research Council, Durban, South Africa; 7 Australian Institute of Health Innovation, Macquarie University, Sydney, Australia; 8 School of Tropical Medicine and Global Health, Nagasaki University, Nagasaki, Japan; 9 Department of Mathematical Sciences, Korea Advanced Institute of Science and Technology, Daejeon, Republic of Korea

Correction to: *Scientific Reports* 10.1038/s41598-019-53838-3, published online 29 November 2019

A supplementary file containing Fig S1 was omitted from the original version of this Article. This has been corrected in the HTML version of the Article; the PDF version was correct at time of publication.

