# Supplementary material for: Malaria predictions based on seasonal climate forecasts in South Africa: A time series distributed lag nonlinear model
Source: Sci Rep. 2019 Nov 29;9:17882. doi: 10.1038/s41598-019-53838-3 (PMC6884483; doi:10.1038/s41598-019-53838-3)
Supplement: Supplementary file 1 — Supplementary information [file 41598_2019_53838_MOESM1_ESM.pdf]

Supplemental Information

Malaria predictions based on seasonal climate forecasts in South Africa: A time series distributed lag nonlinear model

Yoonhee Kim[1], J.V. Ratnam[2], Takeshi Doi[2], Yushi Morioka[2], Swadhin Behera[2], Ataru Tsuzuki[3], Noboru Minakawa[3], Neville Sweijd[4], Philip Kruger[5], Rajendra Maharaj[6], Chisato Chrissy Imai[3,7], Chris Fook Sheng Ng[8], Yeonseung Chung[9], Masahiro Hashizume[3,8*]

1 Department of Global Environmental Health, Graduate School of Medicine, The University of Tokyo, Tokyo, Japan,
2 Application Laboratory, Japan Agency for Marine-Earth Science and Technology, Yokohama, Japan,
3 Institute of Tropical Medicine, Nagasaki University, Nagasaki, Japan,
4 Alliance for Collaboration on Climate and Earth Systems Science, Cape Town, South Africa,
5 Department of Health, Limpopo, South Africa,
6 Office of Malaria Research, Medical Research Council, South Africa,
7 Australian Institute of Health Innovation, Macquarie University, Sydney, Australia,
8 School of Tropical Medicine and Global Health, Nagasaki University, Nagasaki, Japan,
9 Department of Mathematical Sciences, Korea Advanced Institute of Science and Technology, Daejeon, Republic of Korea.

Table of Contents

Model framework

Root mean square error (RMSE)

Figure S1. The weekly-updated malaria prediction process based on the observed weather data (attached as a separate MOV file for animations).

Figure S2. Receiver operating characteristic (ROC) curves and area under the curve (AUC) results by different levels of thresholds.

Figure S3. Sensitivity analysis for modeling choices.

Figure S4. The 3D plot for the nonlinear and delayed association between malaria and temperature.

Figure S5. Weekly malaria predictions based on seasonal climate forecasts for 2- and 4-week-ahead lead time.

Figure S6. Cumulative interannual malaria predictions based on seasonal climate forecasts by different levels of thresholds for 2-week-ahead lead time and the difference between number of predicted outbreaks and the number of true observed outbreaks.

Figure S7. Cumulative interannual malaria predictions based on seasonal climate forecasts for 4-week-ahead lead time.

Figure S8. Malaria cases and proportions by the source of transmission over time.

Model framework

We used a generalized linear model with a Poisson distribution allowing for overdispersion. A representation of the regression model is given as follows:

$$y_t \sim Poisson\ (\lambda_t)$$

$$\text{Log}[\text{E}(\lambda_t)] = \alpha_0 + f(\boldsymbol{x}_t; \boldsymbol{\beta}) + \sum_{p=1}^{P} s_p(z_{pt}; \boldsymbol{\gamma}_p) + y_{t-1}\theta + \text{year}_t\alpha_1$$

where y_t is the observed malaria cases on week t.

$f(x_t; \boldsymbol{\beta})$ represents a flexible function formulated with cross-basis to describe nonlinear and nonlinearly-delayed association between temperature and malaria. $\boldsymbol{x}_t = (x_0, x_{t-1}, \ldots, x_{t-L})$ is a vector of weekly average temperature on week *t* and over the previous L weeks.

$s_p(z_{pt}; \boldsymbol{\gamma}_p)$ represents flexible functions formulated with a natural cubic spline basis to describe an association between precipitation and malaria. The *p* indicates the averaged period (i.e., the *p=1* corresponds to 4-month and *p=2* corresponds to 1-year). For each p, $z_{pt} = \sum_{l=0}^{L_p} \frac{z_{t-l}}{L+1}$ is the moving average of weekly precipitation on week *t* and over the previous L_p weeks ($L_1 = 15$, $L_2 = 51$).

$y_{t-1}\theta$ indicates a linear function for malaria cases at a preceding week (*t*-1).

α_0 is an intercept.
α_1 is a coefficient for year_t to capture the long-term changes in unmeasured time-varying confounders.

Root mean square error (RMSE)

$$\text{RMSE} = \sqrt{\frac{1}{n}\sum_{i=1}^{n} e_i^2}$$

where $e_i = f_i - y_i$ indicating the difference between the prediction (f_i) and the observation (y_i).

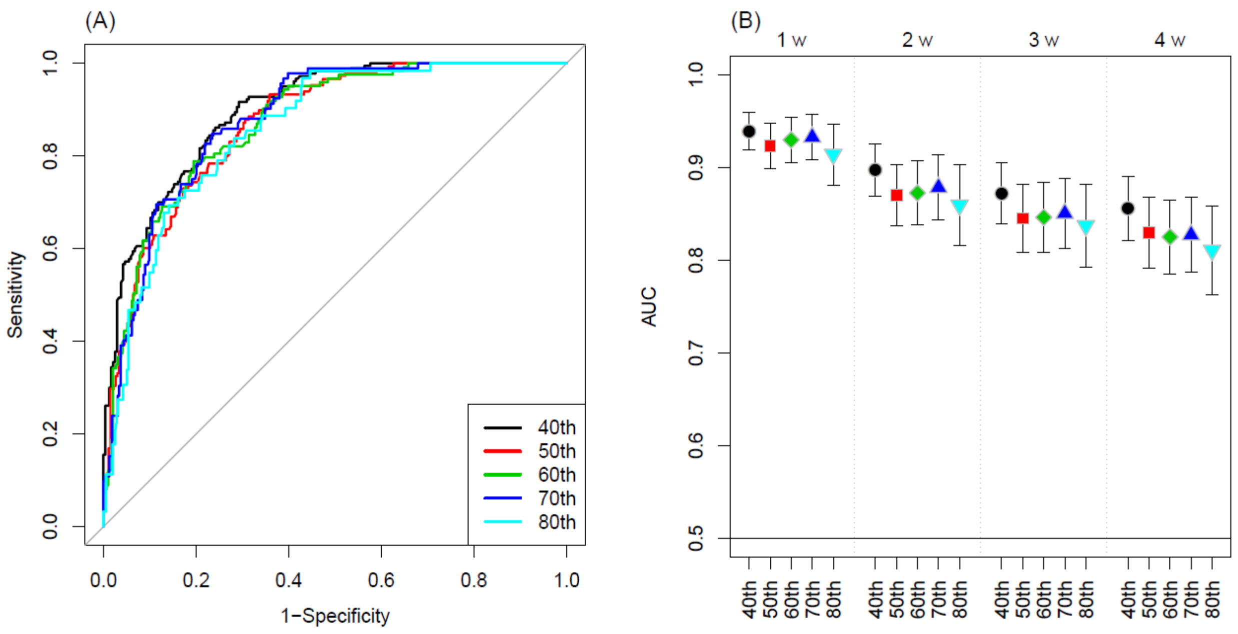

Figure S2. [A] Receiver operating characteristic (ROC) curves and [B] area under the curve (AUC) results by different levels of thresholds from the 40th to the 80th percentiles of the past moving 5-years malaria cases during the endemic season (September–May) in order to determine the optimal level of a threshold to define an outbreak. The AUC values were also calculated from 1-week (1w) to 4-week (4w) ahead predictions.

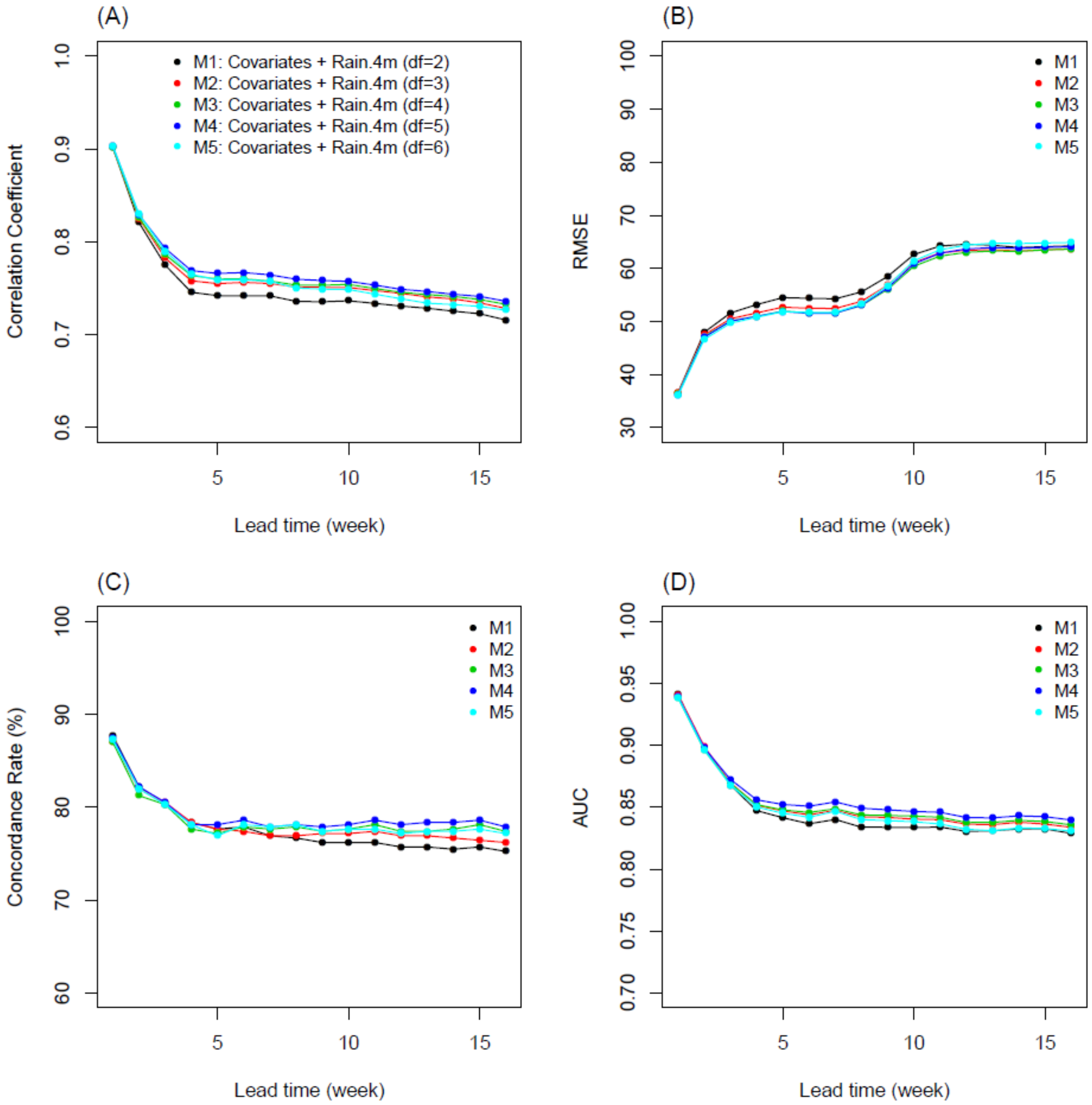

Figure S3-1. Sensitivity analysis for modeling choices. Prediction accuracy measures ([A] correlation coefficients, [B] root mean square error (RMSE), [C] concordance rate, and [D] area under the curve (AUC) of receiver operating characteristic (ROC)) against the lead time (week) by changing the degrees of freedom from 2 to 6 for the exposure-response spline curve of the association between malaria incidence and the shorter-term precipitation (4-month averages).

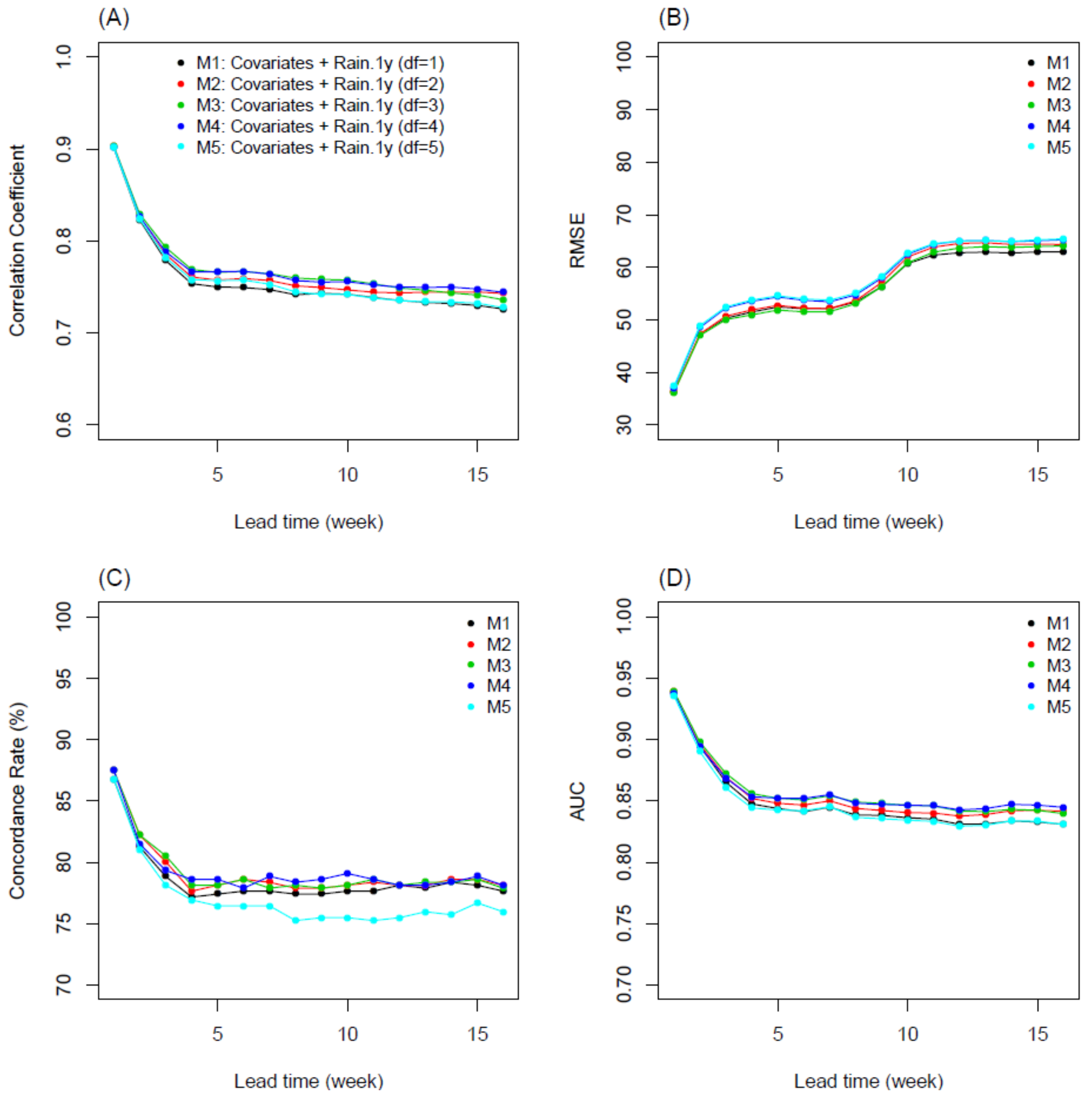

Figure S3-2. Sensitivity analysis for modeling choices. Prediction accuracy measures ([A] correlation coefficients, [B] root mean square error (RMSE), [C] concordance rate, and [D] area under the curve (AUC) of receiver operating characteristic (ROC)) against the lead time (week) by changing the degrees of freedom from 1 to 5 for the exposure-response spline curve of the association between malaria incidence and the longer-term precipitation (1-year averages).

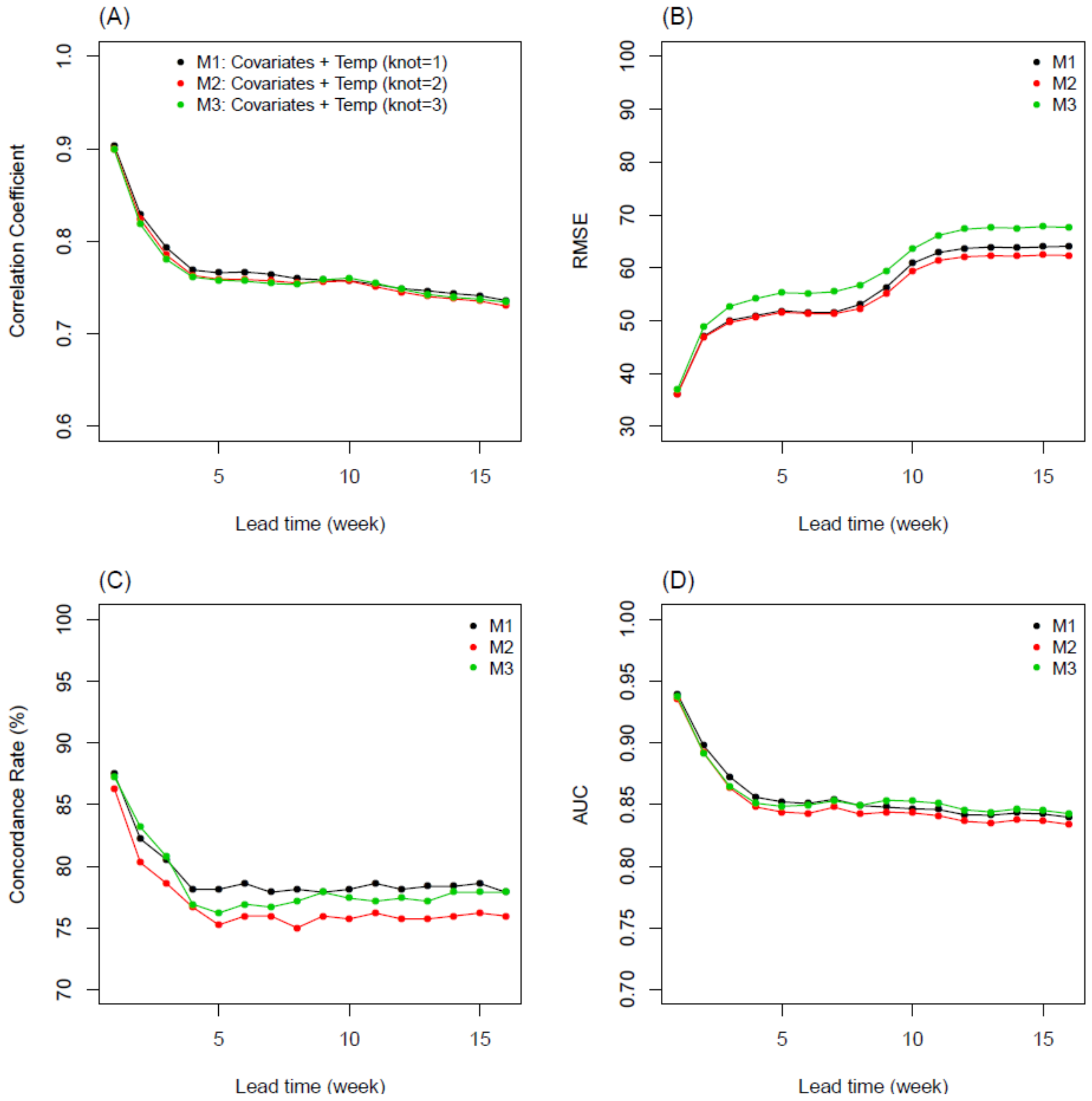

Figure S3-3. Sensitivity analysis for modeling choices. Prediction accuracy measures ([A] correlation coefficients, [B] root mean square error (RMSE), [C] concordance rate, and [D] area under the curve (AUC) of receiver operating characteristic (ROC)) against the lead time (week) by changing the number of internal knots from 1 to 3 for the temperature-malaria association.

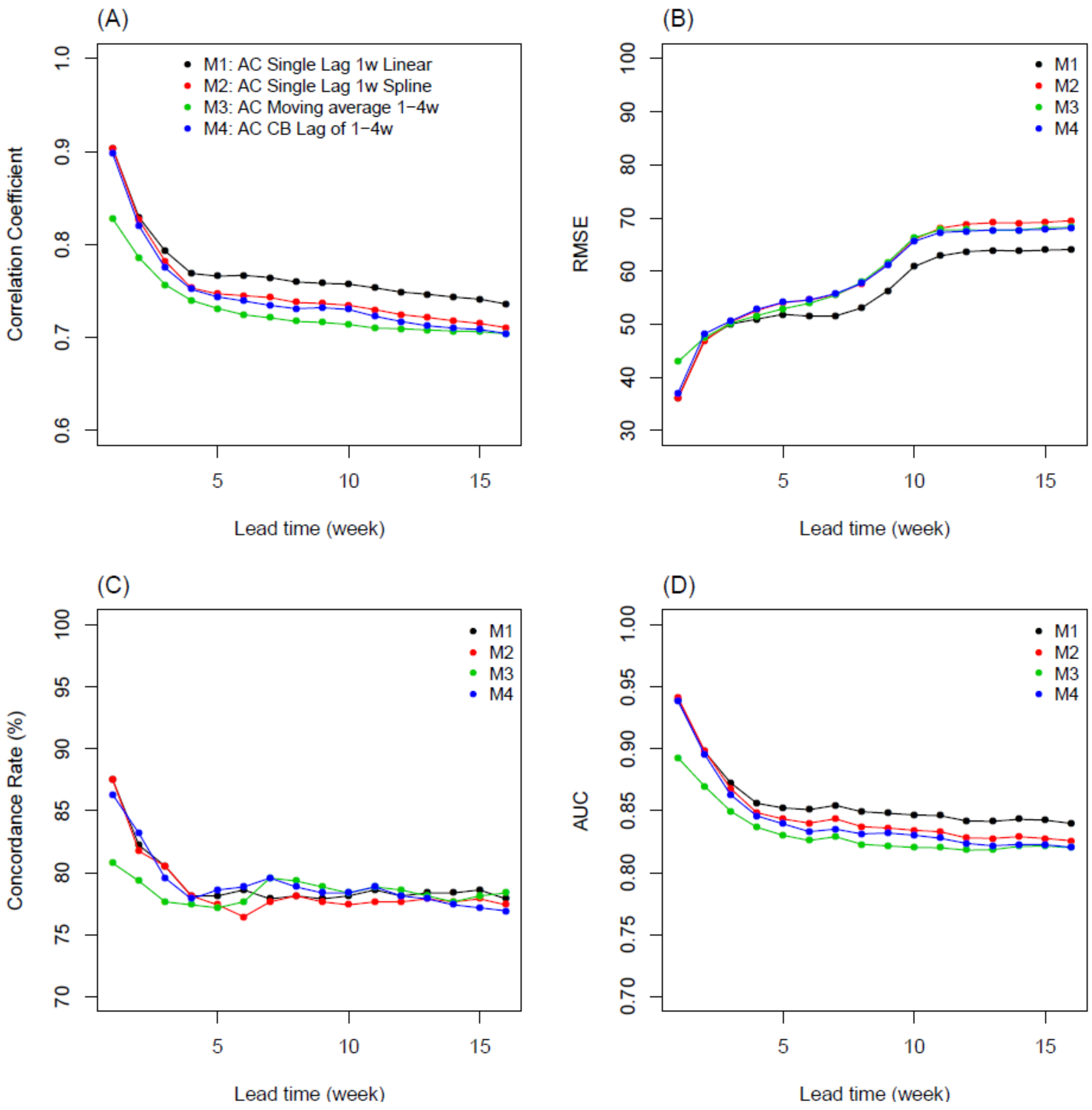

Figure S3-4. Sensitivity analysis for modeling choices. Prediction accuracy measures ([A] correlation coefficients, [B] root mean square error (RMSE), [C] concordance rate, and [D] area under the curve (AUC) of receiver operating characteristic (ROC)) against the lead time (week) by changing the functional forms of the auto-correlation (AC) terms: [M1] AC using single lag of preceding week as a linear term, [M2] AC using single lag of preceding week as a natural cubic spline with 3 df, [M3] AC using moving average of preceding 1–4 weeks as a natural cubic spline with 3 df, and [M4] AC using a cross-basis function including a quadratic B-spline with a knot at the 50th percentile of the preceding 1–4 weeks and a natural cubic spline for the nonlinear distributed lags with a knot at equally-spaced log value.

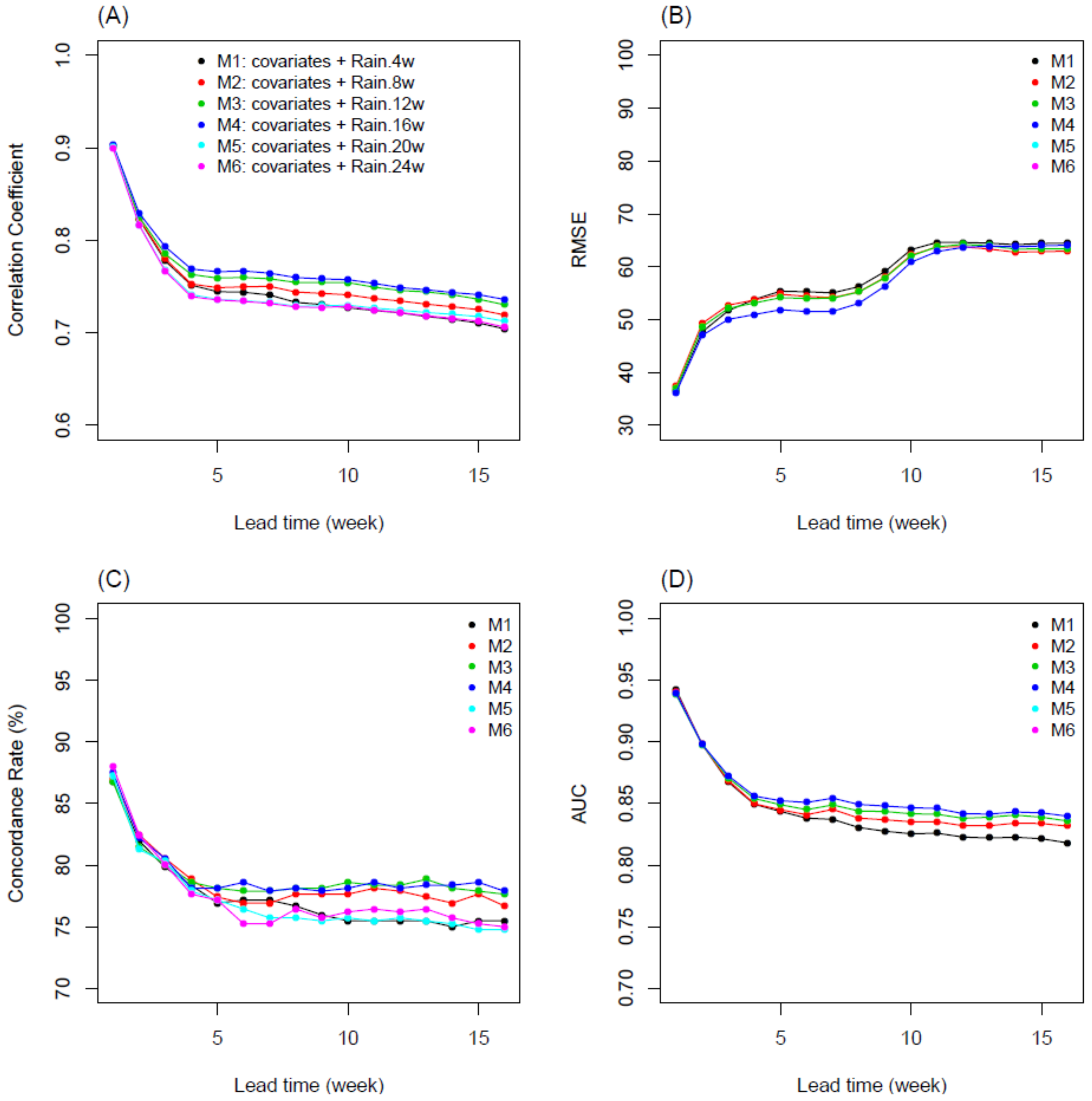

Figure S3-5. Sensitivity analysis for modeling choices. Prediction accuracy measures ([A] correlation coefficients, [B] root mean square error (RMSE), [C] concordance rate, and [D] area under the curve (AUC) of receiver operating characteristic (ROC)) against the lead time (week) by changing the lag periods of the shorter-term precipitation (4-month averages) from 0–4 to 0–24 weeks.

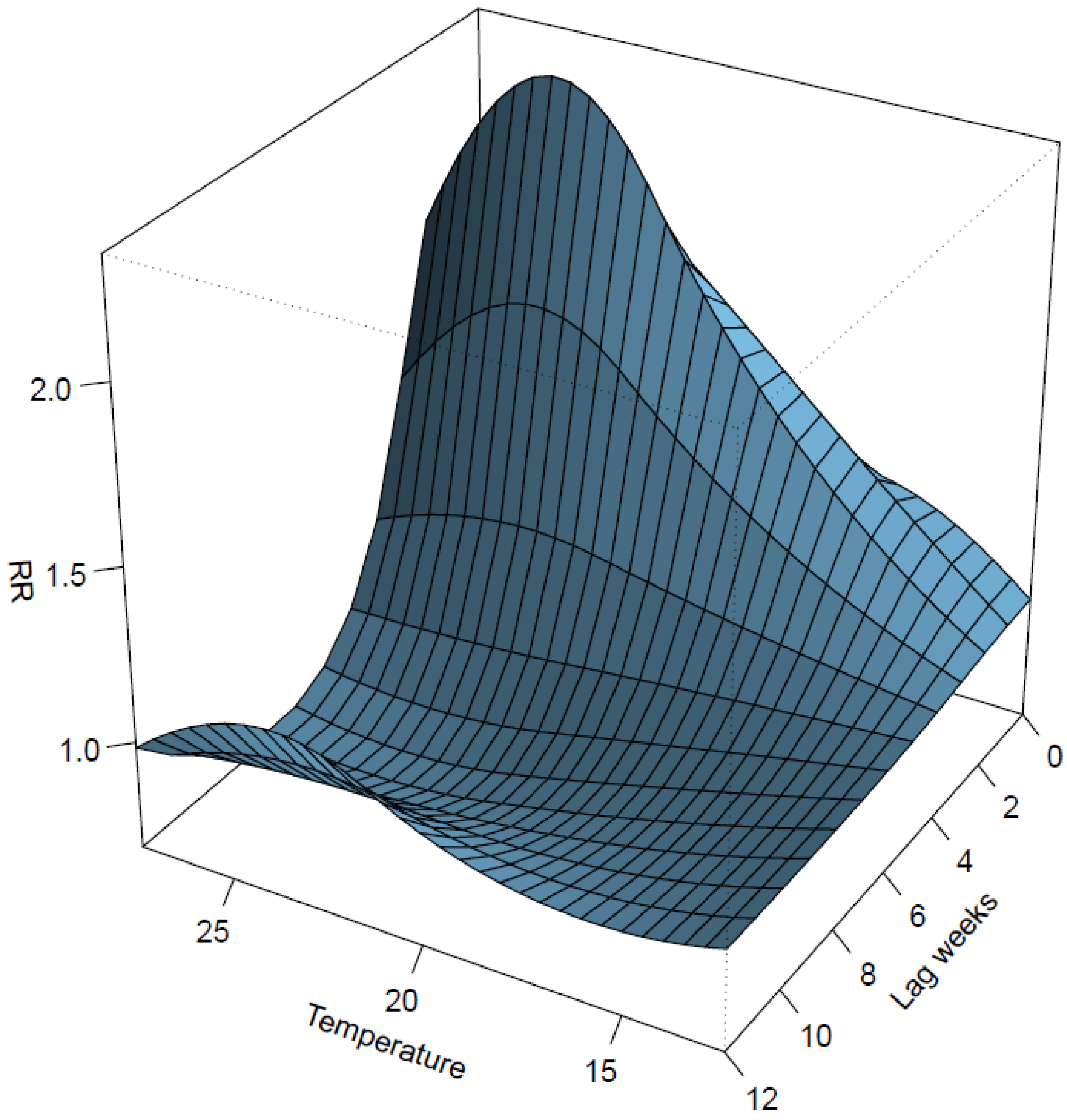

Figure S4. The 3D plot for the nonlinear and delayed association between malaria and temperature.

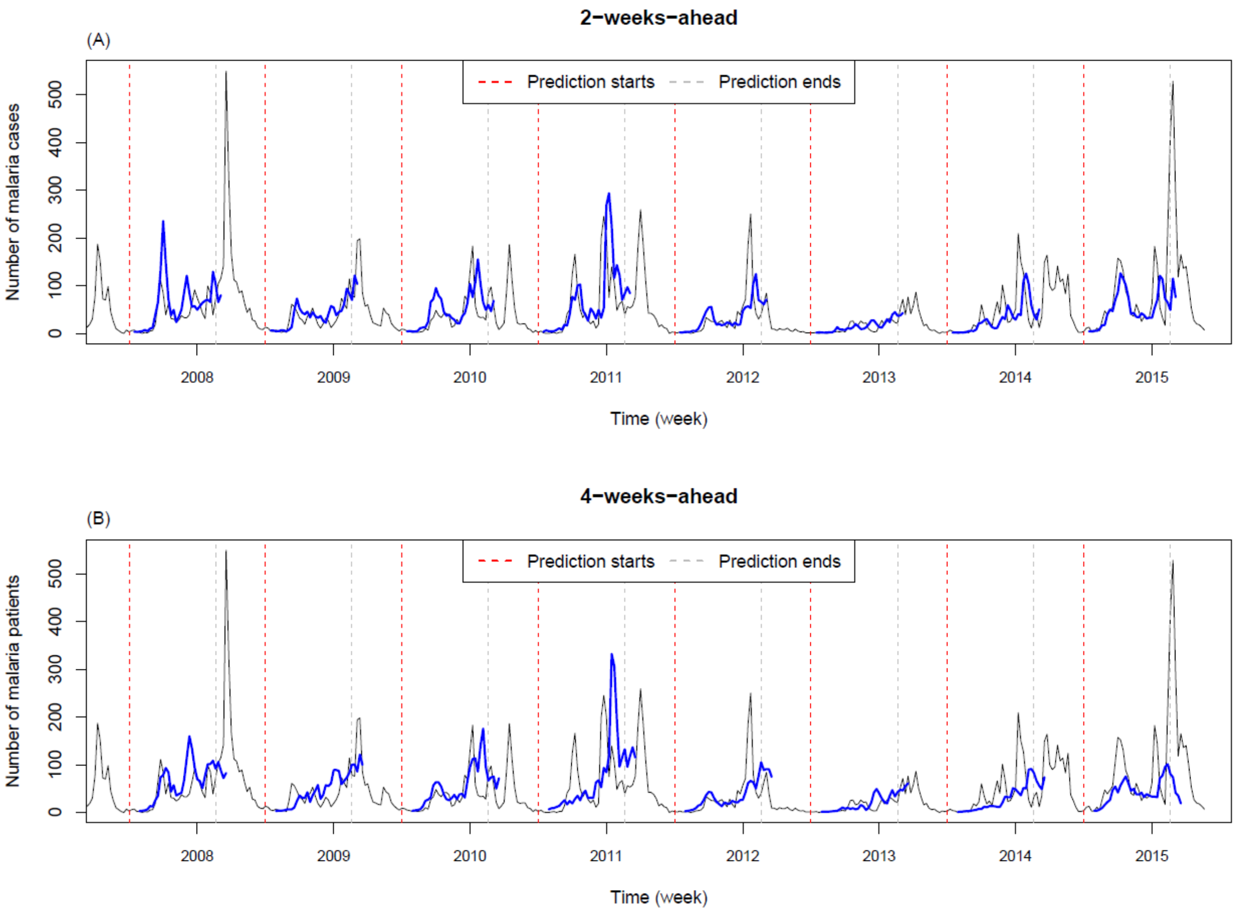

Figure S5. Weekly malaria predictions based on seasonal climate forecasts for 2- and 4-week-ahead lead time.

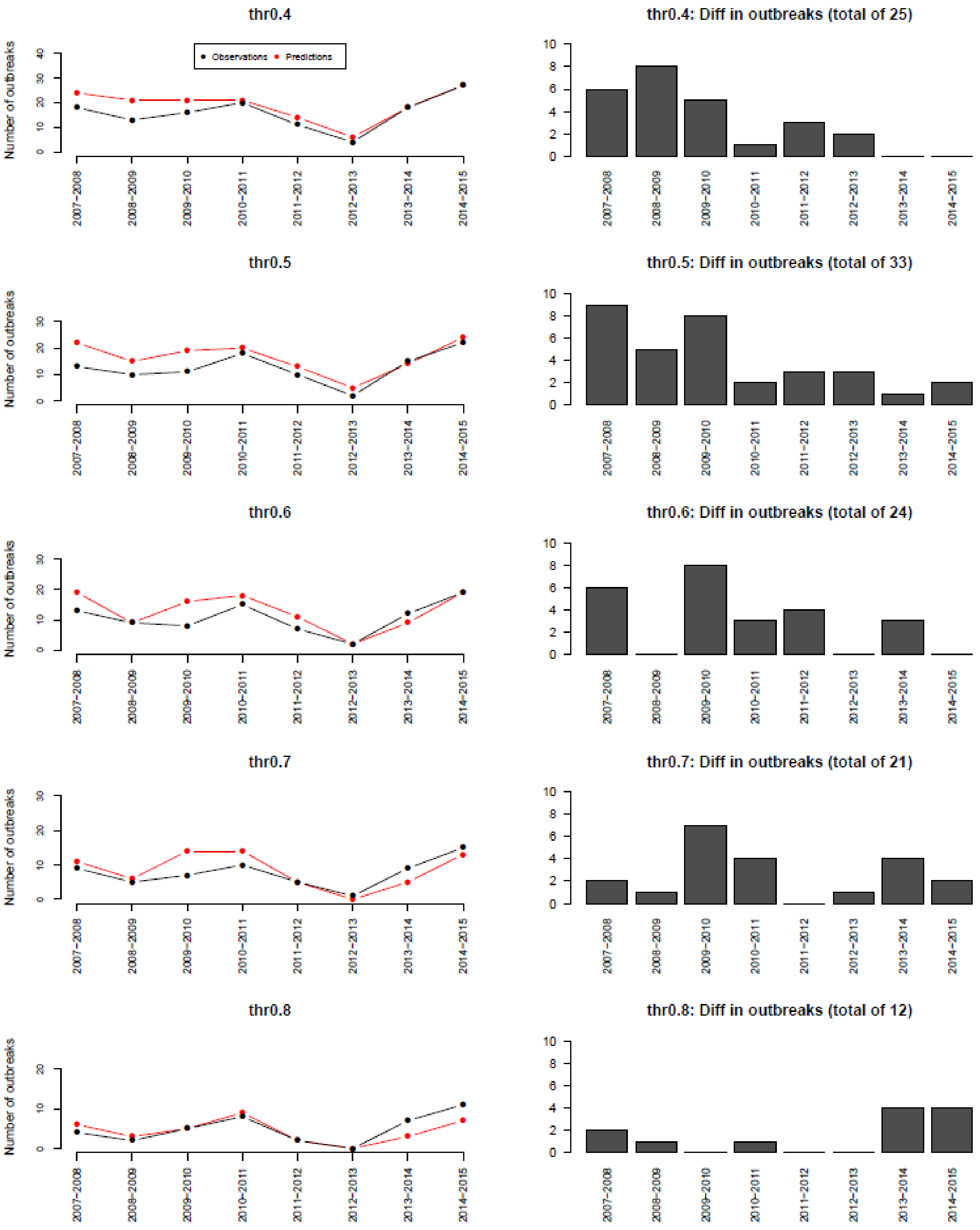

Figure S6. Cumulative interannual malaria predictions based on seasonal climate forecasts by different levels of thresholds for 2-week-ahead lead time (left panel) and the difference between number of predicted outbreaks and the number of true observed outbreaks (right panel).

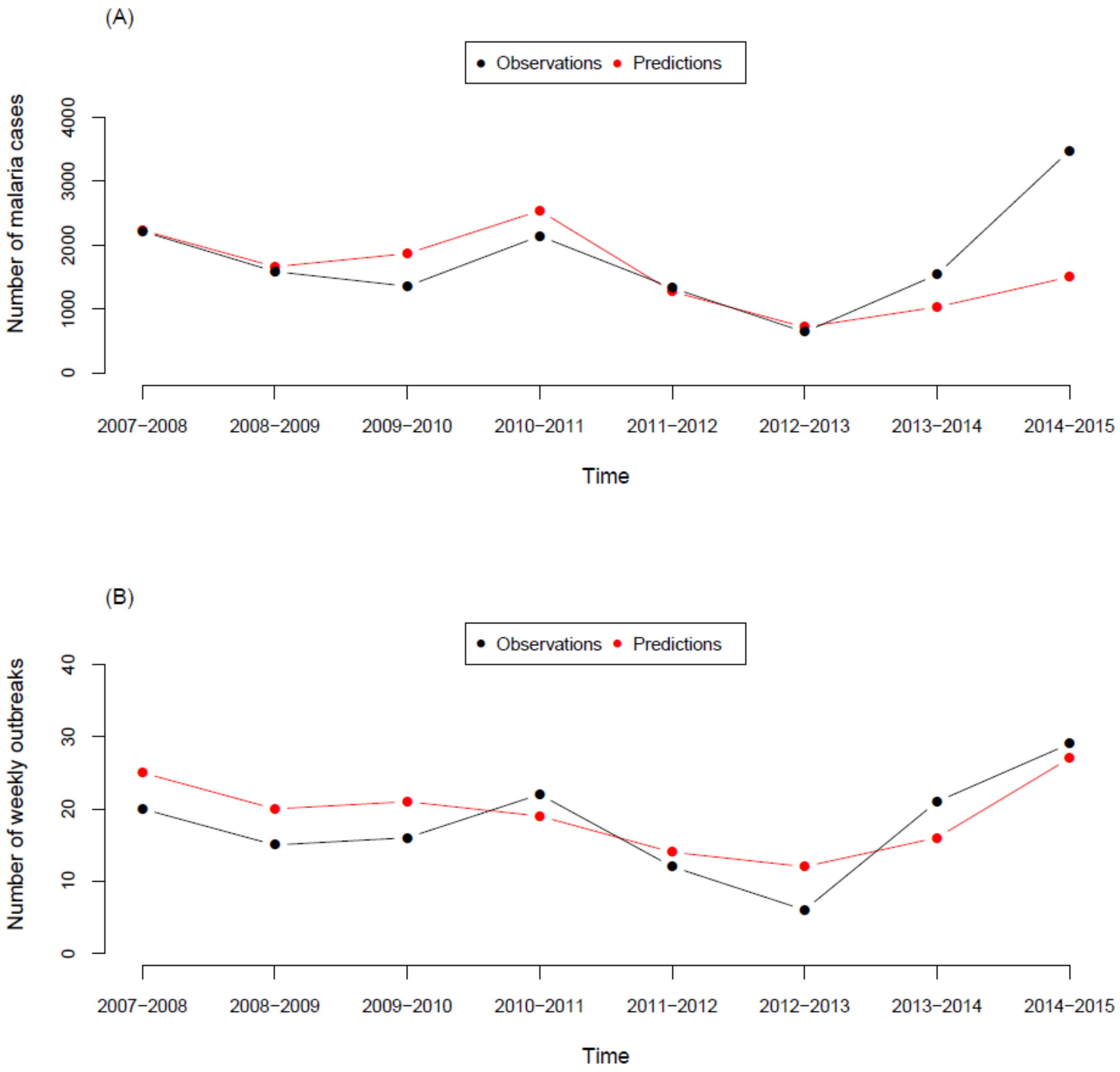

Figure S7. Cumulative interannual malaria predictions based on seasonal climate forecasts for 4-week-ahead lead time.

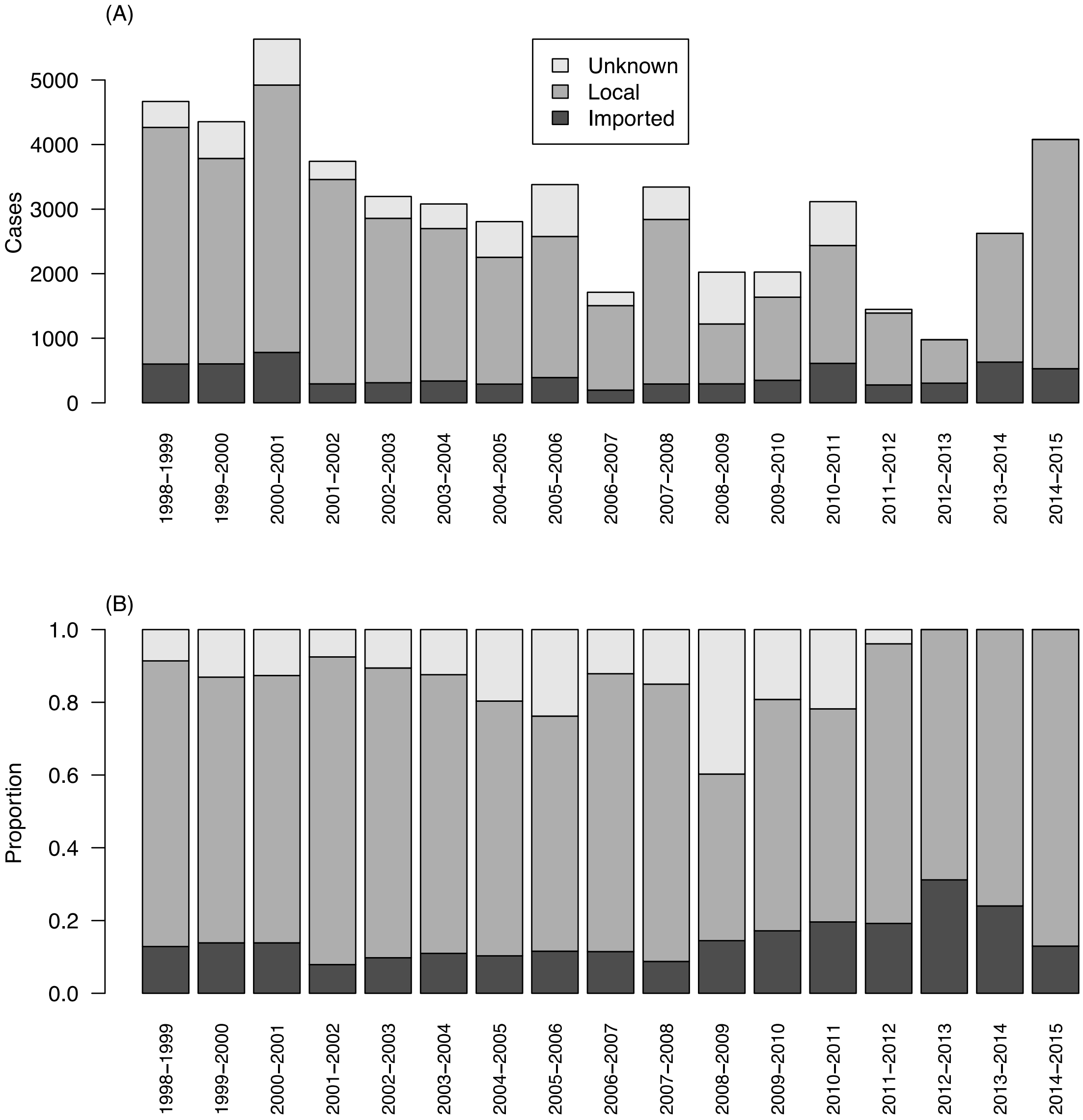

Figure S8. Malaria cases and proportions by the source of transmission over time.
